# A cross-sectional study of symptoms and health-related quality of life in menopausal-aged women in China

**DOI:** 10.1186/s12905-023-02728-y

**Published:** 2023-11-01

**Authors:** Tamlyn A. Rautenberg, Shu Kay Angus Ng, Martin Downes

**Affiliations:** 1 Centre for Applied Health Economics, Griffith University, Brisbane, Australia; 2 Menzies Health Institute Queensland, Brisbane, Australia; 3 Metro North Hospital and Health Service, Brisbane, Australia

**Keywords:** Health state utility values, Premenopausal, Perimenopause, Postmenopausal, Health-related quality of life, Utility, EQ5D5L, Menopause, China

## Abstract

**Objective:**

To measure symptoms and health-related quality of life in a cross-sectional cohort of menopausal-aged women in China.

**Method:**

A cross-sectional survey was conducted in a general population cohort of 2,000 Chinese females over the age of 45 years. Patients completed the Chinese version of the EuroQol-5D five level (EQ5D5L) health-related quality of life instrument via Personal Digital Assistant. Raw scores were converted to utility tariffs using value sets for China. Statistical analysis included Pearson’s chi-square test, z test for multiple comparisons with adjustment by the Bonferroni method, independent-sample t-test, ANOVA, and adjustment by the Tukey method for multiple comparison. Results were considered statistically significant when p < 0.05 and the study was reported according to the STROBE recommendations.

**Results:**

In a cohort of 2000 women, 732 (37%) were premenopausal, 798 (40%) were perimenopausal and 470 (23%) were postmenopausal. Perimenopausal women reported significantly more symptoms (91%) compared to premenopausal (77%) and postmenopausal (81%) women. Health-related quality of life was significantly lower in symptomatic perimenopausal women compared to premenopausal (0.919, p < 0.05) and postmenopausal (0.877, p < 0.05) women. Within each group there was a statistically significant difference between the health-related quality of life of women with symptoms compared to without symptoms.

**Conclusion:**

The perimenopausal phase of menopause is associated with significantly more symptoms and significantly lower HRQoL compared to premenopausal and postmenopausal phases.

**Supplementary Information:**

The online version contains supplementary material available at 10.1186/s12905-023-02728-y.

## Abstract (plain language summary)

*Why is this study needed?*

• It is important to understand the impact of menopausal symptoms on women’s quality of life so that decisions can be made about what healthcare services to provide and what treatments to fund.

• Currently there are only a few older studies investigating menopausal symptoms in the perimenopausal phase, which is characterised by hormone fluctuations and symptoms likely to impact quality of life, and lasts up to four years.

*What is the key problem this manuscript addresses?*

• This research measures symptoms and the impact on quality of life of menopausal women in China.

*What are your main results and what do they mean?*

• Women in the perimenopausal phase are disproportionately affected by symptoms compared to premenopausal and postmenopausal women.

• Perimenopausal women have significantly lower HRQoL compared to premenopausal and postmenopausal women.

• This information is critical to informing pharmacoeconomic decisions about healthcare provision and publicly funded treatments in China.

**Supplementary Information:**

The online version contains supplementary material available at 10.1186/s12905-023-02728-y.

## Introduction

Menopause marks a major event for women and occurs twelve months after the last menstrual period [1]. Importantly, it is preceded by the perimenopause which extends from early through late menopausal transition to early post menopause, lasting up to four years [2]. During perimenopause, women experience hormonal fluctuations associated with vasomotor symptoms (VMS) such as hot flushes, sweating, sleep disturbances, irritability, anxiety, and depression [1, 3, 4]. According to the STRAW criteria, VMS are “likely” and “most likely” during perimenopausal stages − 1 and + 1a respectively [1, 2, 5–8]. This contrasts with the postmenopausal period, when there is low endocrine activity associated with genitourinary syndrome of menopause (GSM) characterised by vaginal dryness, atrophic vaginitis, vulvovaginal pain, pruritus, sexual intercourse pain and urinary problems [1, 5–10].

Currently there are almost 168 million women in China aged 45 to 59 years and considering the mean natural age of menopause (49 years) and the four-year median duration of perimenopause, this translates into a substantial number of women eligible for healthcare services to alleviate menopausal symptoms [1, 4, 11, 12]. Few studies have investigated the burden of symptoms on perimenopausal women in China, with contradictory results, for example Sun et al. reported that symptoms were more severe in postmenopausal women whereas Zhang et al. reported that VMS were more prevalent in perimenopausal women [7, 13].

This is coupled with a lack of evidence on the impact of symptoms on health-related quality of life, again with a focus on perimenopausal women. Measuring HRQoL is key to informing cost utility models (CUM), which are playing an increasingly important role in decision making in China [14]. The current pharmacoeconomic guideline recommends CUM as the preferred method to evaluate the impact of competing technologies [14]. The key requirement for a CUM is HRQoL, specifically health state utility values (HSUV) measured by the preferred, validated and widely used five level EQ5D (EQ5D5L) questionnaire [14]. The EQ5D5L version is an expansion of the EQ5D3L version and a Chinese format with value set for China is available [15].

Currently there is only one study reporting HSUV in China performed a decade ago and unlikely to represent the current situation [16]. Liu et al. examined the relationship between menopause and HSUV in premenopausal versus postmenopausal Chinese women in rural Fangshan, Beijing China [16]. The cross-sectional study measured utility in a cohort of 1351 women of which 656 were premenopausal, 133 were menopausal and 562 were postmenopausal [16]. Noteworthy is that HSUV in the perimenopausal group is not reported. The absence of HRQoL for menopausal women more broadly outside of China is highlighted in a review by Valentzis et al. (2017) which reported that out of five CUM [17–21] three [18, 19, 21] used the same HSUV data from a prior study from Zethraeus [22, 23]. The prior study by Zethraeus measured HRQoL in 104 women almost three decades ago and showed that Menopausal Hormone Therapy improved HRQoL associated with mild symptoms (0.18 to 0.26) and severe symptoms (0.42 to 0.50) using time trade-off and rating scale respectively [23]. The fourth CUM by Ylikangas (2007) used data from a clinical trial in Finland using the 15D instrument in a sample of 210 and 58 respondents at 6 and 9 years respectively [20, 22, 24]. The fifth CUM by Salpeter (2009) used a utility multiplier from a range of sources ranging from 1.0 for older cohorts and 1.07–1.21 for younger cohorts. The multipliers were derived from a range of sources and the methods used to synthesise the data are not described [17]. This lack of recent health state utility data contributes to uncertainty in CUM.

In the absence of published HSUV, a health economist developing a CUM is faced with the option of using suboptimal secondary evidence, making informed model assumptions, or performing primary research [25]. In the case of the latter, timelines and resources may render the face-to-face collection of EQ5D5L data impractical, especially in a large country like China. Research using digital technology offers a promising alternative to time-consuming, resource intense paper-based research [26]. The role of smartphones in the collection of research data is constantly increasing and a range of studies have shown the advantages of using smartphone for data collection [27–31]. Importantly, results are comparable whether collected by paper-based version or smartphone with higher response rates with smartphone versus paper-based versions [32].

In view of the above, the objective of this research was to measure symptoms and health state utility values in a cross-sectional cohort of menopausal-aged women in the general population of China, with the purpose of parameterising a cost utility model and informing healthcare services in China. We hypothesised that women in the perimenopausal age group experiencing symptoms would have the greatest negative impact on HRQoL.

## Method

The study was approved by Griffith Ethics GU Ref No: 2020/389. A commercial license was purchased from EuroQoL and CAHE (Centre for Applied Health Economics, Griffith University) was specified as third-party user. Screening questions included age, gender, uterine status (intact or absent), date of last menses, regularity of menses, symptoms and HRQoL. The screening questions and EQ5D5L were scripted and coded into the survey platform and piloted internally to test the survey on the digital platform, check logic and ensure all questions were comprehensible with no errors in survey flow (n = 20). The screening questions and EQ5D5L were programmed in personal digital assistant (PDA) format for smartphone and underwent two rounds of validation via EuroQoL. A contract research organisation administered the survey via panel data. No formal recruitment was undertaken, the panels were activated and around 30,000 females in the age group of 45 + were invited to the survey link via SMS or WeChat. All participants were provided with a Participant Information Sheet approved by Griffith ethics committee, which described the study and informed participants that their information would be used for research and publication and their responses would remain anonymous. All participants providing informed consent proceeded to the screening questions. Chinese females aged 45 years and over were eligible to participate. All panel respondents were asked the screening questions first, and if eligible, went on to answer EQ5D5L. A general population cohort accruing the first 2000 females over 45 years was used. Respondents received 1.4 USD each via digital wallets of WeChat or Alipay. Three stages of screening occurred: complete questions, passing screening questions and quality control criteria. We took multiple steps to ensure the highest quality of data. Initially we checked cookies, recorded, and blocked repeat internet protocol addresses and checked digital fingerprint (we blocked any participant who completed the survey in the preceding 48 h). We added duplicate questions as checks, for example menopausal regularity was added as first *and* last question with respondents with inconsistent answers disqualified. We also added “trick questions” (5 + 2=, which is a fruit: apple, pear, banana, fish); respondents with incorrect answers were excluded. Finally, we checked the consistency of the answers, for example checking that age corresponded with birth date. Data validation and cleaning were undertaken. No personal information (name, address) was collected and respondents remained anonymous. The research team accessed the back-end of the survey platform and downloaded the completed survey results via excel. The data was stored on a secured Chinese server and disposed at the end of the study (publication of results). EQ5D5L raw scores were converted to utility tariffs using Chinese value sets reported by Luo et al. [15].

Respondents were classified in accordance with the STRAW criteria and the four-year median duration of perimenopause reported by Delamater et al. as follows: respondents with uterus, regular menstruation and reporting last menstrual period up to the study date (December 2020) were classified as premenopausal (reproductive stage); respondents reporting irregular menstruation at the time of the study and up to four years prior to the study were classified as perimenopausal; and women with uterus reporting the date of last menstrual period ≥ four years prior to the study were classified as postmenopausal (see appendix) [2, 3]. Sample size calculations were performed using the G*Power 3.1 statistical software assuming a significance level of 0.05 and a two-tailed test [33]. For dichotomous outcome measures, for example the presence of symptoms, the cohort size of 2000 had more than 98% power to detect a small effect size of 0.1 between three women groups (e.g. pre-, peri-, and postmenopausal) using a chi-square test [34]. For continuous outcome measures, for example utility, the cohort size of 450 in a subgroup analysis (n1 = 70, n2 = 380) had more than 95% power to detect a medium effect size of 0.5 between two women groups (e.g. with and without symptoms) using an independent-cohort t-test [34]. Statistical analyses were performed using IBM SPSS version 27 (IBM, Armonk, NY, USA). Descriptive results were expressed as counts and percentages for categorical variables and as mean and standard deviation for continuous variables. If chi-square test was found significant, further multiple comparisons using z test were performed to test difference in proportions between various menopausal status against perimenopausal with adjustment by the Bonferroni method. An independent-sample t-test was used to compare the utility scores between women with and without symptoms, separately for the pre-, peri-, and postmenopausal groups. An ANOVA was performed to compare the utility scores between women in three different groups (e.g., between the pre-, peri-, and postmenopausal groups). If ANOVA was found significant, further multiple comparisons were performed to test differences among the groups with adjustment by the Tukey method. Results were considered statistically significant when p < 0.05. All methods were carried out in accordance with appropriate guidelines and regulations and are reported in accordance with the STROBE recommendations for reporting of cross-sectional studies [35].

## Results

**Fig. 1:**
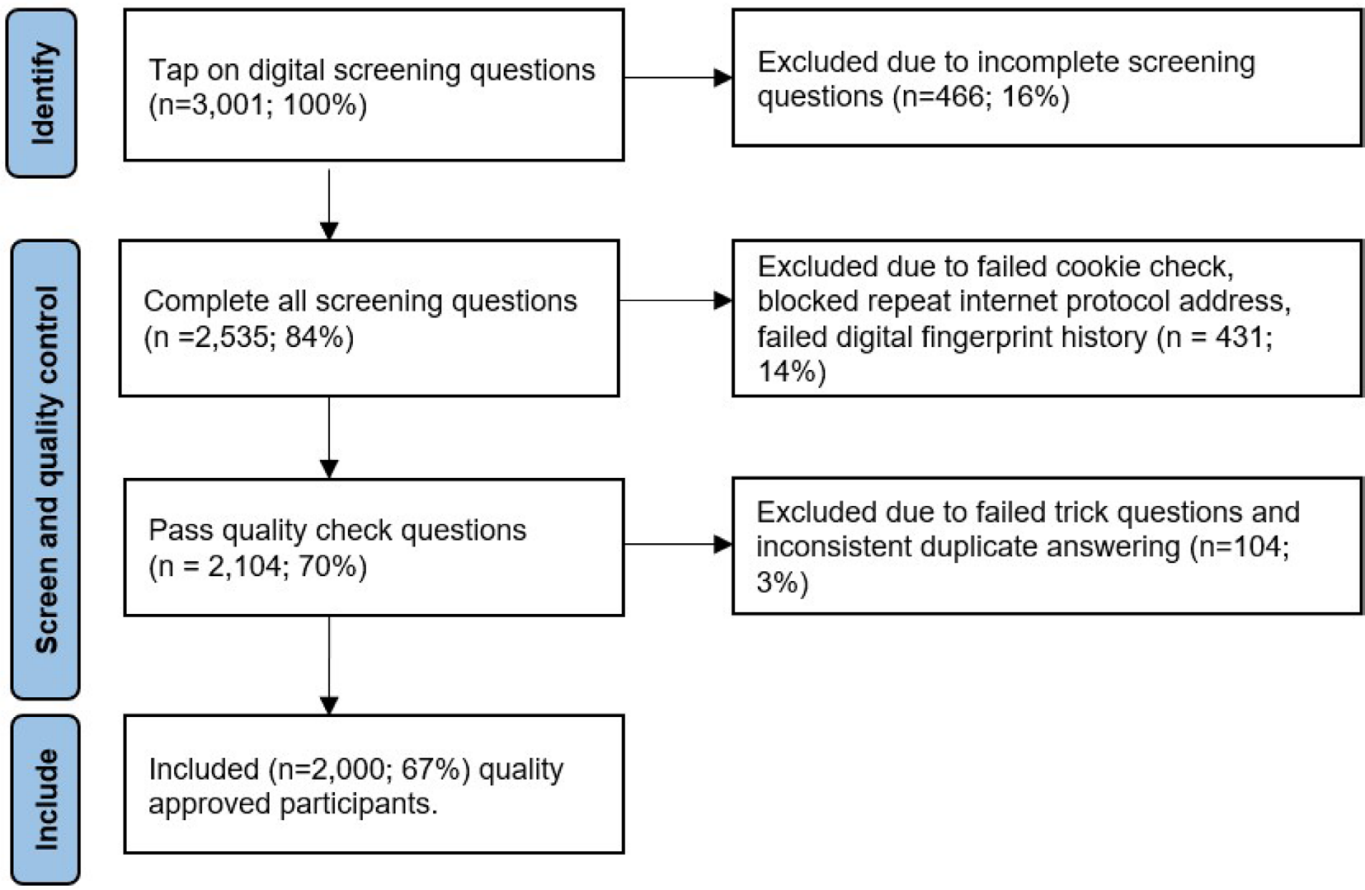
Flow diagram of included cohort

Sample data was collected between 01 October  2020 and 25 December 2020. A total of 3001 tapped on the digital survey of which 466 respondents were excluded due to incomplete screening questions. Of 2535 (84%) who completed the screening questions, 431 (14%) respondents were excluded because they failed the cookie check, digital fingerprint check, or were blocked due to repeat internet protocol address. Out of the remaining 2104 (70%), 104 (3%) respondents were excluded as they failed the trick questions or provided inconsistent answers to the duplicate questions. A final cohort of 2000 (67%) completed all screening and EQ5D5L questions to the required quality and were included in the analysis, as shown in Fig. 1.

The mean age of the cohort was 49 years (range 45–73), with a mean age of 47 years for premenopausal, 49 years for perimenopausal and 53 years for postmenopausal women. There was a significant difference between the age across the groups in keeping with the phases of menopause, therefore it was not appropriate to adjust for age in the analysis of HSUV among the three groups. Respondents were from the East (32%), South-Central (25%), North (26%); Southwest (7%); Northeast (8%) and Northwest (2%) regions. Most respondents had intact uterus (89%). Out of a total cohort of 2000 women, 732 (37%) were classified as premenopausal, 798 (40%) perimenopausal and 470 (23%) as postmenopausal. There were statistically significant differences between the groups for uterine status, EQ5D utility and VAS. Overall, the majority (61%) of women reported being mildly to extremely affected by anxiety/depression and a high proportion were mildly to extremely affected by pain and discomfort (52%). Fewer women reported an effect on mobility (19%), usual activities (18%) and self-care (10%). Comparing the groups, the proportion reporting an effect on EQ5D5L domains was highest in the peri versus pre and post groups for mobility (25% vs. 9%, 22%), self-care (14% vs. 3% and 14%), pain/discomfort (60% vs. 40% and 57%), anxiety/depression (71% vs. 50% and 59%) except for usual activities where post had the highest (24% versus pre and 8% and peri 23%). The characteristics of the cohort are shown in Table 1.

**Table 1:** Characteristics of a general population cohort of menopausal-aged women in China

Characteristics	Total n = 2000	Premenopausal n = 732	Perimenopausal n = 798	Postmenopausal n = 470
Age in years*	49.3 (4.4)	47.4 (2.8)	48.9 (3.2)	52.9 (6.0)
Region				
East	648 (32.4%)	237 (32.4%)	271 (34.0%)	140 (29.8%)
North	510 (25.5%)	192 (26.2%)	206 (25.8%)	112 (23.8%)
Northeast	162 (8.1%)	61 (8.3%)	59 (7.4%)	42 (8.9%)
Northwest	39 (2.0%)	18 (2.5%)	14 (1.8%)	7 (1.5%)
South Central	502 (25.1%)	173 (23.6%)	194 (24.3%)	135 (28.7%)
Southwest	139 (7.0%)	51 (7.0%)	54 (6.8%)	34 (7.2%)
Uterus*				
With	1775 (88.8%)	708 (96.7%)	715 (89.6%)	352 (74.9%)
Without	225 (11.3%)	24 (3.3%)	83 (10.4%)	118 (25.1%)
EQ5D utility score*	0.893 (0.119)	0.929 (0.090)	0.867 (0.129)	0.881 (0.126)
EQ5D domain (mild to severe)				
Mobility*	371 (18.6%)	68 (9.3%)	200 (25.1%)	103 (21.9%)
Self-care*	207 (10.4%)	25 (3.4%)	115 (14.4%)	67 (14.3%)
Usual activities*	358 (17.9%)	57 (7.8%)	186 (23.3%)	115 (24.5%)
Pain/discomfort*	1046 (52.3%)	294 (40.2%)	482 (60.4%)	270 (57.4%)
Anxiety/depression*	1217 (60.9%)	370 (50.5%)	570 (71.4%)	277 (58.9%)
EQ5D VAS percent*	78.7 (12.4)	82.1 (10.9)	75.7 (12.9)	78.4 (12.3)

Data are mean (standard deviation) for continuous variables and counts (percentages) for categorical variables

*Significant difference between pre-, peri-, and postmenopausal groups

Our first key finding was that symptomatic perimenopausal women with intact uterus had significantly lower HRQoL (0.864) than the premenopausal (0.919, *p* < 0.05) and postmenopausal (0.877, *p* < 0.05) women using the ANOVA and Tukey tests. There was also a significant difference between symptomatic versus asymptomatic women within each group as shown in Table 2.

**Table 2:** Health state utility values for pre, peri and post groups with uterus according to symptoms and no symptoms

Group (n)	With symptoms utility (n)	With no symptoms utility (n)	Mean difference (95% CI)	Within group difference with symptoms vs. no symptoms *p*-value
Premenopausal (708)	0.919 (544)	0.977 (164)	−0.058 (−0.070; −0.045)	*p* < 0.05
Perimenopausal (715)	0.864 (649)	0.956 (66)	−0.092 (−0.118; −0.067)	*p* < 0.05
Postmenopausal (352)	0.877 (286)	0.966 (66)	−0.089 (−0.109; −0.068)	*p* < 0.05

N = sample size, *p* = p-value

The second key finding was that a higher proportion of perimenopausal women reported symptoms (91%) compared to premenopausal (77%, difference = 14%, 95% CI = 10–17%) and postmenopausal (81%, difference = 10%, 95% CI = 2–10%) women and this was statistically significant based on z-tests with adjustment by the Bonferroni method. Significantly more perimenopausal women reported nine of the eleven symptoms compared to the premenopausal group (sleep problems, irritability, exhaustion, anxiety, hot flushes, depression, heart discomfort, loss of interest in sexual activity, vaginal dryness). The other two symptoms, although higher, were not statistically significant (joint pain, bladder problems). Significantly more perimenopausal women experienced five of eleven symptoms compared to the postmenopausal group (sleep problems, irritability, anxiety, hot flushes, depression) as shown in Table 3.

**Table 3:** General population cohort of women with uterus and menopausal symptoms

Characteristics	Total n = 1775	Pre n = 708 (40%)	Peri n = 715 (40%)	Peri vs. pre *p*-value	Post n = 352 (20%)	Peri vs. post *p*-value
**Menopausal symptoms**						
Yes	1479 (83%)	544 (77%)	649 (91%)	*p* < 0.05	286 (81%)	*p* < 0.05
No	296 (17%)	164 (23%)	66 (9%)		66 (19%)	
**Vasomotor Symptoms (VMS)**						
Problems sleeping	985 (56%)	351 (50%)	447 (63%)	*p* < 0.05	187 (53%)	*p* < 0.05
Irritability	641 (36%)	211 (30%)	318 (45%)	*p* < 0.05	112 (32%)	*p* < 0.05
Exhaustion	584 (33%)	207 (29%)	258 (36%)	*p* < 0.05	119 (34%)	*p* = 0.46
Anxiety	529 (30%)	177 (25%)	257 (36%)	*p* < 0.05	95 (27%)	*p* < 0.05
Hot flushes	489 (28%)	153 (22%)	245 (34%)	*p* < 0.05	91 (26%)	*p* < 0.05
Joint pain	399 (23%)	140 (20%)	167 (23%)	*p* = 0.10	92 (26%)	*p* = 0.32
Depression	426 (24%)	121 (17%)	232 (32%)	*p* < 0.05	73 (21%)	*p* < 0.05
Heart discomfort	172 (10%)	48 (7%)	84 (12%)	*p* < 0.05	40 (11%)	*p* = 0.85
**Genitourinary Symptoms of Menopause (GSM)**						
LOI in sexual activity	411 (23%)	127 (18%)	195 (27%)	*p* < 0.05	89 (25%)	*p* = 0.49
Bladder problems	59 (3%)	15 (2%)	31 (4%)	*p* = 0.07	13 (4%)	*p* = 0.74
Vaginal dryness	272 (15%)	83 (12%)	131 (18%)	*p* < 0.05	58 (17%)	*p* = 0.46

LOI, loss of interest. Data are counts (percentages). P−values were calculated based on z−tests with adjustment by the Bonferroni method for multiple comparisons

Comparing perimenopausal versus premenopausal women (with uterus), HRQoL was significantly lower for three symptoms having no or little impact on daily life (sleep problems, exhaustion, anxiety); six symptoms having moderate impact on daily life (sleep problems, irritability, exhaustion, hot flushes, heart discomfort, loss of interest in sexual activity) and four symptoms having large impact on daily life (sleep problems, irritability, anxiety, and depression). Comparing perimenopausal versus postmenopausal groups (with uterus), HRQoL was significantly lower for one symptom having large impact on daily life (loss of interest in sexual activity) as shown in Table 4.

**Table 4:** Health state utility values according to symptoms with no/little, moderate and large impact on daily life (respondents with uterus)

Symptom	Pre mean (SD)	Peri mean (SD)	Post mean (SD)	Peri vs. pre mean difference (95% CI)	Peri vs. post mean difference (95% CI)
**Symptoms with no or little impact on daily life**					
Sleep problems	0.949 (0.065)	0.897 (0.114)	0.931 (0.073)	**−0.052** **(−0.081, −0.023)**	−0.034 (−0.072, 0.003)
Irritability	0.913 (0.109)	0.877 (0.104)	0.898 (0.072)	−0.037 (−0.075, 0.002)	−0.021 (−0.072, 0.030)
Exhaustion	0.934 (0.067)	0.904 (0.069)	0.879 (0.100)	**−0.030** **(−0.060, −0.001)**	0.025 (−0.013, 0.063)
Anxiety	0.918 (0.084)	0.849 (0.113)	0.886 (0.075)	**−0.069** **(−0.110, −0.027)**	−0.037 (−0.088, 0.014)
Hot flushes	0.916 (0.078)	0.885 (0.090)	0.870 (0.119)	−0.031 (−0.063, 0.001)	0.014 (−0.023, 0.051)
Joint discomfort	0.909 (0.107)	0.888 (0.109)	0.872 (0.162)	−0.021 (−0.078, 0.037)	0.016 (−0.056, 0.087)
Depression	0.897 (0.123)	0.901 (0.092)	0.887 (0.082)	0.004 (−0.045, 0.054)	0.014 (−0.053, 0.081)
Heart discomfort	0.897 (0.099)	0.846 (0.151)	0.858 (0.142)	−0.051 (−0.133, 0.031)	−0.012 (−0.104, 0.080)
LOI sexual activity	0.911 (0.065)	0.881 (0.115)	0.880 (0.110)	−0.030 (−0.068, 0.009)	0.001 (−0.041, 0.043)
Bladder problems**	0.912 (0.033)	0.773 (0.138)	0.889 (0.104)	−0.139 (−0.340, 0.062)	−0.115 (−0.294, 0.063)
Vaginal dryness	0.904 (0.060)	0.871 (0.103)	0.883 (0.152)	−0.033 (−0.085, 0.018)	−0.012 (−0.072, 0.047)
**Symptoms with moderate impact on daily life**					
Sleep problems	0.905 (0.074)	0.863 (0.108)	0.860 (0.126)	**−0.043** **(−0.068, −0.018)**	0.003 (−0.027, 0.033)
Irritability	0.896 (0.082)	0.838 (0.126)	0.830 (0.163)	**−0.059** **(−0.095, −0.022)**	0.007 (−0.039, 0.054)
Exhaustion	0.900 (0.080)	0.842 (0.117)	0.855 (0.121)	**−0.058** **(−0.093, −0.023)**	−0.013 (−0.052, 0.026)
Anxiety	0.880 (0.085)	0.844 (0.120)	0.853 (0.132)	−0.036 (−0.072, 0.001)	−0.009 (−0.053, 0.036)
Hot flushes	0.907 (0.092)	0.825 (0.151)	0.842 (0.124)	**−0.083** **(−0.136, −0.030)**	−0.017 (−0.085, 0.051)
Joint discomfort	0.863 (0.102)	0.813 (0.149)	0.841 (0.124)	−0.050 (−0.102, 0.002)	−0.028 (−0.086, 0.030)
Depression	0.880 (0.094)	0.833 (0.132)	0.826 (0.147)	−0.047 (−0.099, 0.004)	0.007 (−0.049, 0.063)
Heart discomfort	0.882 (0.060)	0.745 (0.219)	0.818 (0.063)	**−0.136** **(−0.265, −0.008)**	−0.073 (−0.248, 0.102)
LOI sexual activity	0.897 (0.073)	0.827 (0.136)	0.818 (0.159)	**−0.071** **(−0.131, −0.011)**	0.008 (−0.060, 0.077)
Bladder problems	0.861 (0.123)	0.818 (0.114)	0.755 (0.133)	−0.043 (−0.184, 0.098)	0.064 (−0.098, 0.226)
Vaginal dryness	0.890 (0.103)	0.850 (0.130)	0.868 (0.102)	−0.040 (−0.109, 0.028)	−0.019 (−0.094, 0.057)
**Symptoms with large impact on daily life**					
Sleep problems	0.889 (0.093)	0.813 (0.151)	0.859 (0.131)	**−0.076** **(−0.121, −0.030)**	−0.046 (−0.096, 0.004)
Irritability	0.882 (0.084)	0.792 (0.157)	0.848 (0.136)	**−0.091** **(−0.152, −0.029)**	−0.056 (−0.123, 0.011)
Exhaustion	0.869 (0.094)	0.818 (0.132)	0.804 (0.194)	−0.051 (−0.118, 0.016)	0.014 (−0.066, 0.094)
Anxiety	0.881 (0.084)	0.796 (0.163)	0.835 (0.136)	**−0.085** **(−0.155, −0.015)**	−0.039 (−0.127, 0.050)
Hot flushes	0.865 (0.129)	0.792 (0.225)	0.716 (0.208)	−0.073 (−0.245, 0.099)	0.076 (−0.090, 0.242)
Joint discomfort	0.861 (0.086)	0.778 (0.155)	0.761 (0.162)	−0.083 (−0.179, 0.013)	0.017 (−0.072, 0.106)
Depression	0.891 (0.051)	0.737 (0.188)	0.820 (0.145)	**−0.154** **(−0.230, −0.078)**	−0.084 (−0.180, 0.013)
Heart discomfort**	0.811 (0.074)	0.674 (0.180)	0.622 (0.201)	−0.137 (−0.380, 0.106)	0.052 (−0.103, 0.206)
LOI sexual activity	0.899 (0.090)	0.844 (0.100)	0.919 (0.066)	−0.055 (−0.114, 0.005)	**−0.075** **(−0.145, −0.004)**
Bladder problems**	0.946 (0.076)	0.733 (0.127)	0.679 (0.274)	−0.213 (−0.650, 0.224)	0.054 (−0.328, 0.435)
Vaginal dryness	0.906 (0.081)	0.828 (0.117)	0.830 (0.165)	−0.078 (−0.169, 0.014)	−0.002 (−0.100, 0.096)

p-value based on ANOVA with adjustment by the Tukey method for multiple comparisons. The perimenopausal group has a significantly lower utility compared to the premenopausal group based on ANOVA with adjustment by the Tukey method for multiple comparisons. Bolded values p < 0.05 for peri versus pre groups **insufficient sample size to detect significant differences. No statistically significant differences were found between the peri and post groups

## Discussion

Our study provides insight into a quota of menopausal-aged women drawn from the general population in China. In keeping with our hypothesis, perimenopausal women experienced significantly more symptoms and had significantly lower HRQoL compared to premenopausal and postmenopausal women. The HRQoL of women in our study was notably lower (0.893) than the general population (0.962) for 40–49-year-old females in urban China [36]. The mean utility of premenopausal women (0.929) was more closely aligned to the general population and declined in the postmenopausal group (0.881) [versus 0.954 (0.933; 0.975) for 50–59-year-olds] [36].

To the best of our knowledge the only other study measuring HSUV in menopausal women using EQ5D3L in China is by Liu et al., however it compares the HSUV of premenopausal (0.810) versus postmenopausal (0.800) women and does not report the HSUV for perimenopausal women [16]. The study findings are in agreement with a review of the impact of menopausal transition on HRQoL more broadly [37]. Matthews et al. reported that, based on twelve cross sectional studies, perimenopause is associated with more somatic symptoms, however none of the studies used the EQ5D5L nor were they conducted in China [37]. Other studies use a range of generic and disease specific instruments as described by Zollner and Matthews and are therefore not directly comparable to our study [37, 38]. Zöllner’s review of HRQoL instruments concluded that of the eight instruments reviewed, none were found to capture all relevant aspects of HRQoL and treatment. In Taiwan a cohort of 734 premenopausal women was followed up for 2 years and HRQoL was assessed with SF36 and HADS with no effect [39]. Hess enrolled 732 women of menopausal age in a GP practice in USA and measured HRQoL with the RAND-36 and found that physical health was poorer in late peri- and early post- versus premenopausal women. The mental health component was lowest in late peri- versus early post-, late post- and premenopausal women [40]. A recent study evaluated the influence of education on perimenopausal symptoms and HRQoL using the disease specific World Health Organisation Quality of Life BREF Questionnaire. Higher education corresponded to better HRQoL in perimenopause women in 1632 treatment-naïve women attending an outpatient clinical in Hangzhou, Zhejiang Province, China [1]. Adding further complexity is the timing of the studies over different phases of menopause, and comparison between different time periods during menopause [37]. Finally, other studies use different drugs formulations [39–44]. One study evaluated the prevalence of screening-detected depression and the association of depression with HRQoL in community-dwelling postmenopausal women living in three Asian countries including China [45]. In 336 women in Chengdu or Kunming metropolitan areas (mean age 59 years), HRQoL was measured using EQ5D, however the health state utility values are not reported [45]. Our findings differ from the study by Zhang that showed that menopausal symptoms were more severe in postmenopausal women [13]. Zhang et al. highlight that this may be selection bias due to the study group comprising women who “traveled from all over China to one, specialized center;” so this is cohort more affected by symptoms. Furthermore, a generic or recognised disease specific instrument was not used to measure HRQoL [13].

Our research findings should be interpreted in the context of the strengths and weaknesses of our study. In keeping with other studies collecting patient reported outcomes using electronic methods, one of the key strengths of our study was the timely data collection in a large cohort with low resource demands [46]. Another unique advantage of smartphone format was the flow and layout of the questionnaire meant that each question needed to be answered before respondents could proceed to the next question, as a result there are no missing values in respondents who got the end of the EQ5D5L questions (i.e., those with complete surveys). Response rates were high (2000/3001 = 67%) compared to other studies likely due to digital accessibility [40]. Screening questions and EQ5D5L were in local Chinese language. In our study we used the EQ5D5L rather than the EQ5D3L used by Liu, which is more sensitive to changes in HRQoL [16]. Importantly, results of administering the paper-based version of EQ5D are comparable to electronic version [32]. The cohort is a representative sample of the general population based on quota and no randomisation occurred, although it is the basis for the statistical tests. The study is descriptive, and due to the cross-sectional design cannot evaluate causality. Many of the symptoms measured may occur independent of menopause and due to the nature of the study there was no way to determine whether that was the case in our quota. Our study did not measure detailed socio-demographic characteristics such as chronic diseases, and lifestyle factors like physical activity, smoking and alcohol consumption which could confound results. It is likely that biological and social confounders are contributing factors to the HRQoL measured in our study. We cannot correlate our findings with clinical hormone levels therefore we cannot confirm the link between menopause, symptoms, and HRQoL. A recent study reported that hypomnesia was the third most common symptom experienced by 71% of postmenopausal and 66% of perimenopausal women however hypomnesia is not measured in our study [13]. The respondents were restricted to women with digital literacy with access to smartphone and are therefore likely to have better education and higher socioeconomic status. Since our data was collected during the COVID pandemic the status of our participants and their responses may have been affected by the outbreak. Although our findings have high internal validity; we caution against generalising our findings to the broader population. Despite the limitations, the findings are useful and provide insight into the current status of menopausal-aged women in China. Future research would benefit from studies measuring the causal relationships between menopause, symptoms and HRQoL.

## Conclusion

The perimenopausal phase of menopause is associated with significantly more symptoms and significantly lower HRQoL compared to premenopausal and postmenopausal phases.

### Electronic supplementary material

Below is the link to the electronic supplementary material.

Supplementary Material 1

## Data Availability

All data generated or analysed during this study are included in this published article and its supplementary information file.

## Code Availability

(software application or custom code): All data generated or analysed during this study are included in this published article and its supplementary information file.
