# Supplementary material for: A cross-sectional study of symptoms and health-related quality of life in menopausal-aged women in China
Source: BMC Womens Health. 2023 Nov 1;23:563. doi: 10.1186/s12905-023-02728-y (PMC10621238; doi:10.1186/s12905-023-02728-y)
Supplement: Supplementary file 1 — Supplementary Material 1 [file 12905_2023_2728_MOESM1_ESM.docx]

Supplement

A cross-sectional study of symptoms and health-related quality of life in menopausal-aged women in China.

Rautenberg TA^1,2,3^, Ng SK^1^, Downes M^1^

1.Centre for Applied Health Economics, Griffith University, Brisbane, Australia

2. Menzies Health Institute Queensland, Brisbane, Australia

3. Metro North Hospital and Health Service, Brisbane, Australia

Classification of women in the study.

**Premenopausal**

Respondents with uterus, regular menstruation and reporting last menstrual period up to the study date (December 2020) were classified as premenopausal (reproductive stage).


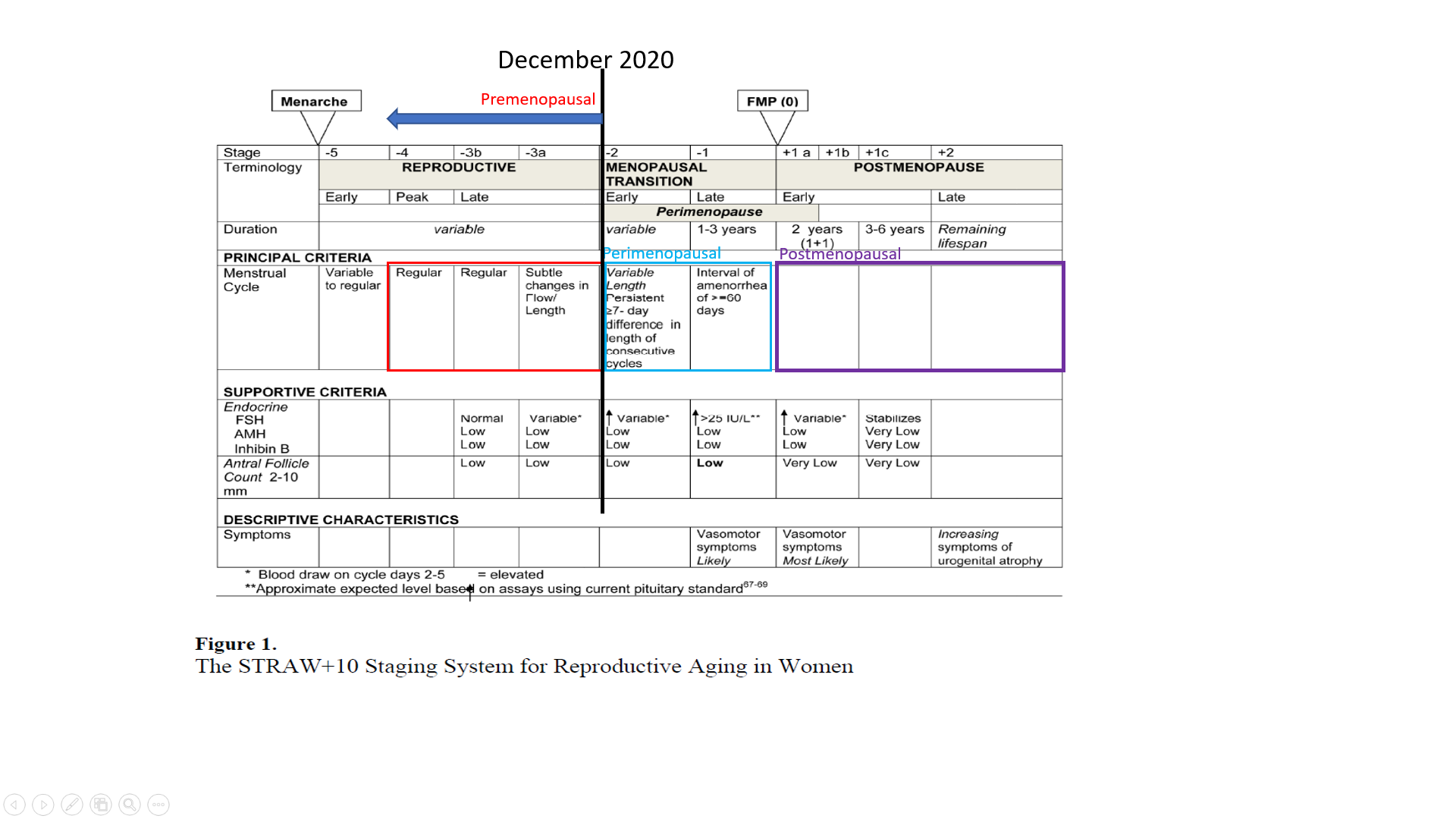


Harlow 2021 STRAW +10 Staging System for Reproductive Aging in Women

**Perimenopausal**

Respondents reporting irregular menstruation at the time of the study and up to four years prior to the study were classified as perimenopausal (in keeping with median duration of 4 years perimenopause reported by Delamater).


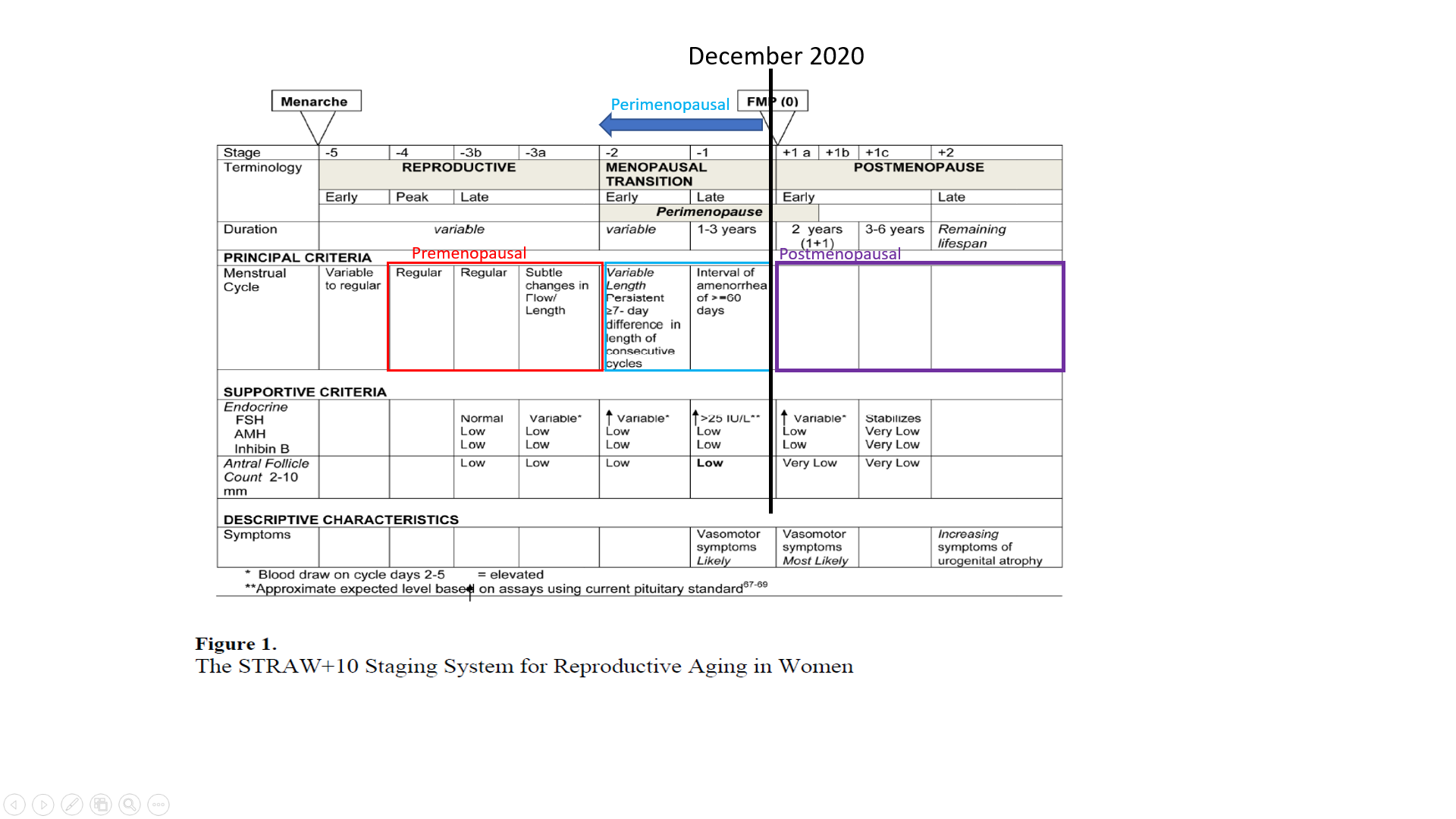


Harlow 2021 STRAW +10 Staging System for Reproductive Aging in Women

**Postmenopausal**

Women with intact uterus reporting the date of last menstrual period ≥ four years prior to the study data collection date were classified as postmenopausal.
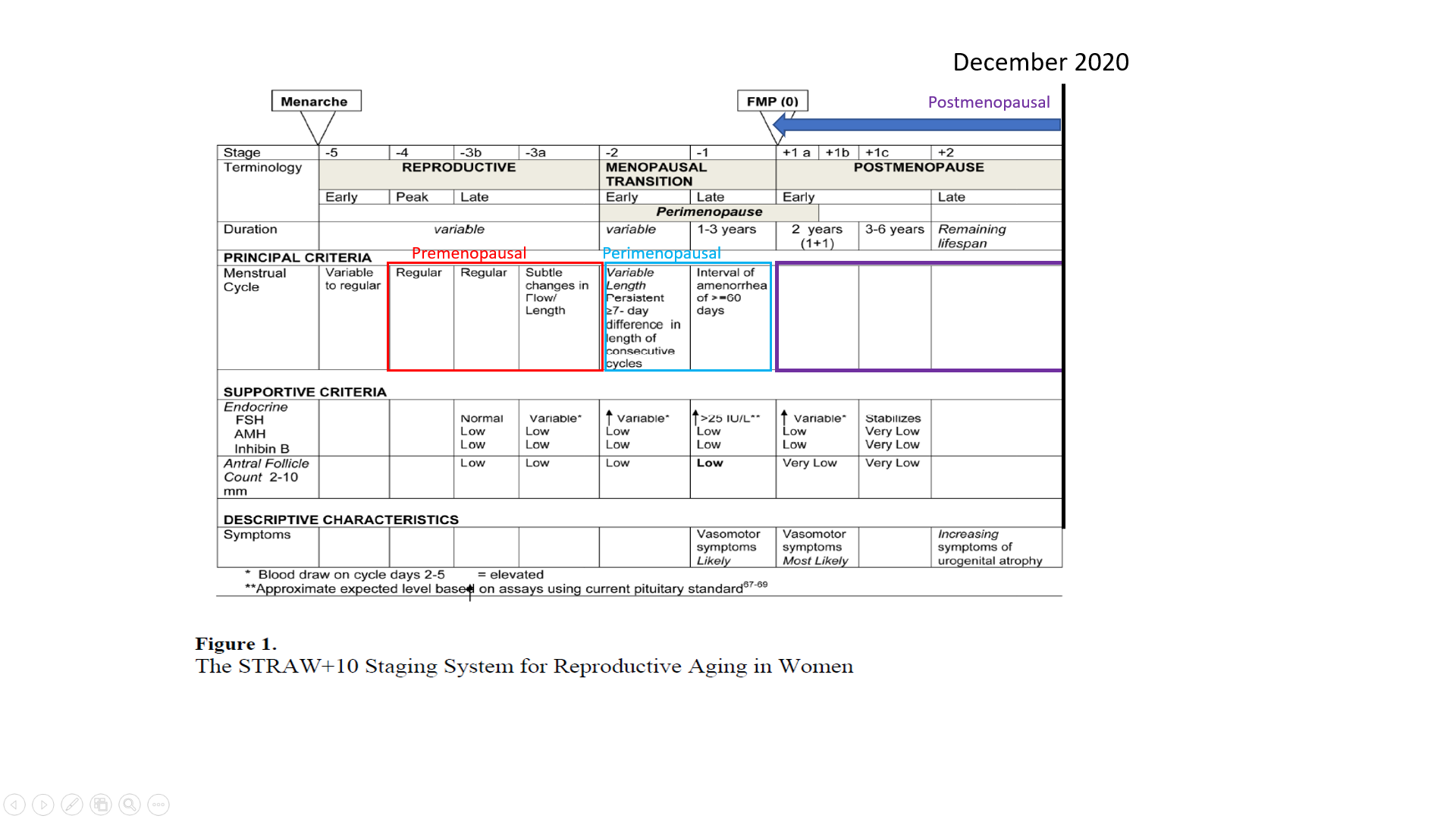


Harlow 2021 STRAW +10 Staging System for Reproductive Aging in Women
